# The impact of injuries study. multicentre study assessing physical, psychological, social and occupational functioning post injury - a protocol

**DOI:** 10.1186/1471-2458-11-963

**Published:** 2011-12-31

**Authors:** Denise Kendrick, Claire O'Brien, Nicola Christie, Carol Coupland, Casey Quinn, Mark Avis, Marcus Barker, Jo Barnes, Frank Coffey, Stephen Joseph, Andrew Morris, Richard Morriss, Emma Rowley, Jude Sleney, Elizabeth Towner

**Affiliations:** 1 Division of Primary Care, Tower Building, University Park, NG7 2RD Nottingham, UK; 2 Centre for Transport Studies, Dept of Civil, Environmental and Geomatic Engineering, UCL, Gower Street, London WC1E 6BT, UK; 3 Nursing, Midwifery & Physiotherapy Department, Nottingham University Hospitals NHS Trust, Queen's Medical Centre Campus, Nottingham NG7 2UH, UK; 4 Division of Psychiatry, Institute of Mental Health, B Floor, Sir Colin Campbell Building, University of Nottingham Innovation Park, Triumph Road, Nottingham NG7 2TU, UK; 5 Transport Safety Research Centre, Loughborough Design School, Loughborough University, Ashby Road, Loughborough LE11 3UZ, UK; 6 Emergency Department, Nottingham University Hospitals NHS Trust, Queen's Medical Centre Campus, Nottingham NG7 2UH, UK; 7 Sociology & Social Policy Department, University Park, Nottingham NG7 2RD, UK; 8 CLAHRC NDL, Sir Colin Campbell Building, University of Nottingham Innovation Park, Nottingham NG7 2TU, UK; 9 Department of Sociology Faculty of Arts and Human Sciences, University of Surrey, Guildford, Surrey GU2 7XH, UK; 10 Centre for Child & Adolescent Health, University of the West of England, Oakfield House, Oakfield Grove, Clifton, Bristol BS8 2BN, UK

**Keywords:** Unintentional injury, Disability, Psychological morbidity

## Abstract

**Background:**

Large numbers of people are killed or severely injured following injuries each year and these injuries place a large burden on health care resources. The majority of the severely injured are not fully recovered 12-18 months later. Psychological disorders are common post injury and are associated with poorer functional and occupational outcomes. Much of this evidence comes from countries other than the UK, with differing health care and compensation systems. Early interventions can be effective in treating psychological morbidity, hence the scale and nature of the problem and its impact of functioning in the UK must be known before services can be designed to identify and manage psychological morbidity post injury.

**Methods/Design:**

A longitudinal multi-centre study of 680 injured patients admitted to hospital in four areas across the UK: Nottingham, Leicester/Loughborough, Bristol and Surrey. A stratified sample of injuries will ensure a range of common and less common injuries will be included. Participants will complete a baseline questionnaire about their injury and pre-injury quality of life, and follow-up questionnaires 1, 2, 4, and 12 months post injury. Measures will include health and social care utilisation, perceptions of recovery, physical, psychological, social and occupational functioning and health-related quality of life. A nested qualitative study will explore the experiences of a sample of participants, their carers and service providers to inform service design.

**Discussion:**

This study will quantify physical, psychological, social and occupational functioning and health and social care utilisation following a range of different types of injury and will assess the impact of psychological disorders on function and health service use. The findings will be used to guide the development of interventions to maximise recovery post injury.

## Background

Worldwide 5.8 million people die [1] and more than 45 million are moderately or severely disabled following injury each year [2], making injuries responsible annually for 10% of all deaths [1] and 16% of all disabilities [2]. Injuries were the leading cause of preventable years of life lost between the ages of 0 and 74 years in 2005 [3]. The scale of the problem is likely to increase, as injury related deaths are projected to rise by 28% between 2004 and 2030, predominantly due to deaths from road traffic injury [2]. Unintentional injuries also place a large burden on health care resources. They result in more than 11,000 deaths in England and Wales [4], three quarter of a million hospital admissions in England, resulting in more than 3.6 million bed days [5] and 5.8 million Emergency Department (ED) attendances in the UK [6]. Working age adults comprise 40% of unintentional injury deaths, 35% of hospital admissions and 50% of ED attendances [5-7].

Several large prospective studies have examined long-term outcomes of injury [8-25]. These studies have used different populations, different outcome measurement tools and different data collection methods, follow-up periods and timings of follow-up. Despite these differences a consistent picture emerges that most severely injured patients are not fully recovered 12 to 18 months post injury [10,11,26], and even amongst less severely injured patients, 45% are not fully recovered 12 months post injury [16]. Furthermore, functioning may deteriorate in the longer term for some of those with severe injuries [13].

Recent reviews [27-30] suggest the prevalence of psychological disorders post injury is high and that these may be associated with poorer functional and occupational outcomes [10,26,31]. A review of psychiatric morbidity after motor vehicle collisions found the most commonly reported disorders were depression (21% to 67% across studies), anxiety (4% to 87% across studies), driving phobia (2% to 47% across studies) and PTSD (0% to 100% across studies) [30]. A second systematic review reported post traumatic injury rates of PTSD ranging across studies from 2-30%, depression from 6-42%, with up to half of those with PTSD also having co-morbid depression. Anxiety disorders were reported to range from 4-24%, with up to 60% of those with PTSD also having co-morbid anxiety disorders. Specific travel phobias for those injured in motor vehicle collisions were reported to range from 4-29% [28]. A third review found rates of PTSD to range from 2% to 50% across studies [29].

Few large prospective studies have measured psychological morbidity following injury in the UK. Psychiatric disorders were found to be common in injured male ED attenders in the short (48% at 6 weeks) and medium (43% at 6 months) term [32]. A second study of road traffic injured ED attenders found 8% had developed PTSD by 3 months, and nearly a quarter had psychiatric complications at one-year [33]. A third study by the same authors in a similar study population found 36% reported psychological problems at 3 months and 32% at 1 year, with PTSD being reported in 23% at 3 months and 17% at 1 year [16]. The generalisability of the findings of these studies to wider population groups and to those suffering a range of injuries is unclear. Although a recent large UK prospective study of injury related disability has been undertaken [34], this does not measure psychological outcomes.

Evidence suggests that screening tools may be useful in health care settings for identifying those at risk [35], and early interventions can be effective in treating psychological morbidity following injury. Individual trauma-focused cognitive behavioural therapy (TFCBT), stress management and group TFCBT are effective in the treatment of PTSD [36] and pharmacotherapy, particularly selective serotonin reuptake inhibitors (SSRIs) are effective in reducing symptoms of PTSD and associated depression [37]. There is also limited evidence that psychosocial interventions may not help prevent physical, psychological and social disability post injury but that an intervention based on complex collaborative care may do so [38]. Current UK guidelines propose that heath and social care workers should understand the psychological impacts of trauma and, as an immediate response, offer practical, social and emotional support. In addition the guidance supports the use of TFCBT and the use of antidepressants [39]. A health service model has been proposed for identifying those who may benefit from such interventions [28], but in order to design such a service, the prevalence of such morbidity and its impact on functioning and costs, must be known. The importance of, and need for qualitative research in establishing the needs of injured patients, areas of unmet need, gaps in service provision and barriers and facilitators to accessing services for the purposes of informing service provision has also been highlighted [40-44]. Further exploration of the experiences of service users, carers and service providers in the UK is required in order that services can be designed which will maximise recovery post injury.

### Aims

The aim of the study is to measure and characterise physical, psychological, social and occupational outcomes post unintentional injury and identify service use, gaps in service provision and information needs, and barriers and drivers to accessing services.

### Objectives

The objectives of this study are to:

• Measure physical, psychological, social and occupational outcomes post unintentional injury

• Measure health and social care provision, use and cost

• Quantify the impact of psychological problems on recovery from a range of unintentional injuries

• Identify service use, gaps in service provision and information needs, and barriers and drivers to accessing services from the perspective of those with injuries, their carers and service providers.

## Methods/Design

### Participants

This is a longitudinal multi-centre study with a nested qualitative study recruiting participants admitted to hospital with a wide range of unintentional injuries from 4 UK study centres (Nottingham, Leicester/Loughborough, Bristol, and Surrey). A stratified sampling frame (Table 1) will be used to guide recruitment to ensure a range of common and less common injuries will be included and to allow comparison with other studies of injury morbidity [20,22,34]. Participants will be recruited in Emergency Departments (EDs), on hospital wards, in outpatient departments (OPDs), or by post following hospital discharge. Participants with upper and lower limb injuries and those with multiple injuries, and their carers along with representative service providers, will be eligible for, and recruited by post to the qualitative study.

**Table 1:** Sampling frame for recruitment by age, gender, and injury type^i^

	Male	Male	Male	Female	Female	Female
	**16-24**	**25-59**	**60-70**	**16-24**	**25-59**	**60-70**

Facial fracture, eye injury	10	10	10	10	10	10

Spine, vertebrae injury	10	10	10	10	10	10

Internal organ injury	10	10	10	10	10	10

Upper extremity fracture	10	10	10	10	10	10

Upper extremity, other injury	10	10	10	10	10	10

Hip fracture	< 10	10	10	< 10	10	10

Lower extremity fracture	10	10	10	10	10	10

Lower extremity, other injury	10	10	10	10	10	10

Superficial injury, open wounds	10	10	10	10	10	10

Burns	10	10	< 10	10	10	< 10

Poisoning	10	10	10	10	10	10

Other injury	10	10	10	10	10	10

Total by age and gender	110	120	110	110	120	110

Total by gender		340			340	

^i ^Where there are < 10 expected participants in any cell of the sampling frame, attempts will be made to recruit as many people as possible, but these cells have not been included in the total number of participants

### Centres for recruitment

Recruitment will be undertaken in NHS Trusts at the 4 study centres: Nottingham University Hospitals NHS Trust Queens Medical Centre campus, Leicester Royal Infirmary, Bristol Royal Infirmary, Frenchay Hospital (Bristol), and the Royal Surrey County Hospital.

### Exclusion/Inclusion Criteria

Inclusion criteria: patients aged between 16 and 70 years, who are admitted to hospital in one of the participating centres following an unintentional injury, which occurred up to 3 weeks prior to the date of recruitment, and who are able to give consent will be eligible to participate in the longitudinal study. Participants with upper and lower limb injuries and those with multiple injuries and their carers and service providers will be eligible for the qualitative study.

Exclusion criteria: patients will be excluded if they are below the age of 16 and above the age of 70 at the time of their injury, do not have an address (due to inability to follow-up these patients), are not admitted to hospital, do not allow access to their medical notes, or are unable to give consent. Patients with significant head injuries (defined as loss of consciousness, amnesia or a Glasgow coma scale of < 15 at presentation) will be also excluded due to the difficulty of distinguishing between the sequelae of even mild head injury and psychological morbidity [45,46].

### Measures

At baseline (day of recruitment to the study) participants will be asked to complete a questionnaire covering circumstances surrounding their injury, socio-demographic and occupational details, health status, quality of life and social and occupational functioning in the 4 weeks prior to their injury. The following standardised tools will be used: the Alcohol Use Disorders Identification Test (AUDIT) [47], the Drug Abuse Screening Test (DAST) [48], the Hospital Anxiety and Depression Scale (HADS) [49], an adaptation of the Accident Fear Questionnaire (AFQ) [50], the EQ5D [51], the HUI-3 [52], the Work Limitations Questionnaire [53] and the Social Functioning Questionnaire [54]. Participants will also undergo a shortened structured clinical diagnostic interview (SCID) [55] to determine pre-injury psychological morbidity. A small incentive (£2 high street gift voucher) will be given to participants on receipt of completed questionnaires.

At 1 month, 2 months, 4 months, and 12 months post injury [56], participants will be asked to complete follow-up questionnaires covering whether they are still affected by their injury, perceptions of recovery, factors that helped or hindered recovery, health and social care resource use, time off work and litigation and compensation. In addition, they will be asked to complete the standardised tools used at baseline as well as the Impact of Event Scale (IES) [57], the Trauma Screening Questionnaire (TSQ) [35], the Changes in Outlook Scale (CIO), [58], the Crisis Support Scale (CSS) [59], the List of Threatening Events (LTE) [60] and a visual analogue pain scale. Participants scoring above threshold values on the AUDIT, DAST, HADS, IES or TSQ will be contacted to undertake a shortened SCID administered face-face or by telephone, containing questions related only to the tool(s) for which they scored above the threshold value. Follow-up questionnaires will be administered by post, phone, or via email depending on participant preference. Non-responders will be followed up by 2 mailed questionnaires and/or telephone reminders. A small incentive (£2 high street gift voucher) will be given to participants on receipt of completed questionnaires.

Data will be extracted from the medical records to allow injury severity scoring using the Abbreviated Injury Scale (AIS) [61]. Socio-economic status will be based on area deprivation scores derived from the postcode of residence using the 2010 Index of Multiple Deprivation [62]. Aggregated data on age group, gender and injury type will be collected for a 6 month period from patients who do not consent to the study to explore the generalisability of findings.

#### Qualitative study

Semi-structured interviews will explore participants experience of their injury and their post injury care including factors that facilitate or hinder recovery such as access to healthcare and social support and issues surrounding the effects of litigation and compensation. Interviews will be conducted in the patient's homes or by telephone and will be audio recorded and transcribed. A maximum of 48 interviews will be undertaken in total across the 4 study centres. Maximum variation sampling will be used to obtain a sample of injured participants with injuries of varying types and severities, varying degrees and types of psychological morbidities, levels of deprivation, social support, age and gender. Interviewed participants will also identify carers and representative service providers to be interviewed. A maximum of 32 carers will be interviewed to explore perceptions of the recovery process and factors that facilitate or hinder recovery from a carer's perspective. A minimum of 32 service providers will be interviewed to explore factors that facilitate or hinder recovery from the perspective of people who deliver services. Additional interviews will be undertaken with managers or commissioners of services where these exist.

#### Ethical Considerations

The study has multi-centre research ethics committee approval from the Nottingham Research Ethics Committee 1 (number: 09/H0407/29).

#### Analysis

Baseline characteristics of participants will be described using frequencies and percentages for categorical variables and means (and standard deviations (SD)) or medians (and inter-quartile ranges (IQR)) depending on the shape of their distributions, for continuous variables.

At each follow-up time-point the prevalence of binary and categorical physical, psychiatric, social and occupational outcomes will be described using percentages (and 95% Confidence Intervals). Scores for standardised scales will be described using means (and SDs) or medians (and IQRs) depending on the shape of their distributions. Changes from baseline pre-injury health status, quality of life, social and occupational functioning will be calculated and described using means (and SDs) or medians (and IQRs) depending on the shape of their distributions. As the use of a multi-centre study design will affect the precision of estimates of prevalence and means, this will be accounted for in the estimation of 95% confidence intervals.

Random-effects generalised linear models will be used to quantify the association of psychological morbidity with EQ5D, HUI, work limitations, time off work due to injury and social functioning. This analysis will use repeated measures of both the outcomes and the psychological morbidity variables at 1, 2, 4 and 12 months, with participant as a level 2 unit (cluster) and measurement occasion as a level 1 unit, to allow for correlations of measurements within patients. The exposure variables of interest are psychiatric diagnoses, defined as meeting the Diagnostic and Statistical Manual (DSM) criteria for each disorder measured by the SCID. Analyses will be adjusted for study centre (Nottingham, Bristol, Surrey, Loughborough/Leicester). Causal diagrams will be drawn to identify confounders for inclusion and effect mediators for exclusion from models. Follow-up time will also be included in the models. Tests of interaction will be carried out between having a psychiatric diagnosis and confounding variables using likelihood ratio tests, to examine whether any association between having a psychiatric diagnosis and each outcome of interest differs according of the level of the confounding variable. Tests of interaction will also be carried out between psychiatric diagnosis and follow-up time to see whether the associations change with time after injury.

### Checks of assumptions

We will assess multicollinearity through calculation of correlations and VIF values. We will calculate residuals at both levels and assess these for normality; if they do not show an approximately normal distribution then transformations will be applied. We will compare results with and without excluding observations with large standardised residuals (< - 3 or > 3 standard deviations from the mean of a normal or normalised random variable).

### Additional analyses

Factors associated with psychiatric diagnoses will be explored using univariate and multivariate random-effects generalised linear models This analysis will use repeated measures of the psychiatric diagnosis variables at 1, 2, 4 and 12 months, with participant as a level 2 unit (cluster) and measurement occasion as a level 1 unit, to allow for correlations of measurements within patients. The main outcome variable will be a binary variable for any psychiatric diagnosis, Further analyses for specific diagnoses will only be undertaken where the sample size is sufficient.

### Economic Analysis

The economic analysis will be carried in accordance with the statistical analysis outlined above: random-effects generalised linear models will be used to quantify the association of psychological morbidity with resource use and costs from the NHS, Personal Social Services (PSS) and societal perspectives. Subject to sufficient statistical power being established a posteriori, resource use and costs will be compared across several a priori sub-groups, including

• With depression/not

• With PTSD/not

• With anxiety disorders/not

• With alcohol misuse disorders/not

• With substance misuse disorders/not

• With travel fear and avoidance

• With 2 or more psychiatric diagnoses (at any time over the follow-up period)

• With previous history psychiatric diagnosis (prior to or at time of injury)

Separate analyses will be undertaken from each of the perspectives. Costs will be derived by assigning unit costs to units of patient-reported resource use; unit costs will be collected from published sources: the BNF; NHS Reference Cost Schedule and PSSRU; ONS [63-66]. This analysis will also estimate the costs attributable to psychological morbidity.

A sub-sample of 100 patients will have their medical records audited to compare with self-reported resource use, using a pre-existing data extraction form. If it appears from the sub-sample that self-reported resource use is biased systematically (i.e. consistently under - or over-reporting resource use), we will model this bias in the sub-sample, then use the model to correct the resource use reported in the full sample. We will conduct sensitivity analyses by comparing the results of the economic analyses using different estimates of resource use.

### Missing data

Missing data will be subjected to sensitivity analysis with respect to the outcome and exposure variables to determine whether it is reasonable to assume missingness at random. If appropriate, we will use multiple imputation to replace missing values at baseline or follow-up. We will compare results with a complete case analysis. If data are not missing at random, either sensitivity to inclusion/exclusion/imputation will be reported, or selection models will be explored.

#### Sample size

680 participants will be recruited, with an estimated 456 (67%) followed up for 1 year. This provides 80% power (alpha = 0.05) to detect differences in the EQ5D, between those with, and without the condition of interest, of between 0.08 (anxiety) and 0.13 (depression) assuming a standard deviation of 0.23 based on population norms [67], or differences in the EQ5D of between 0.10 (anxiety) and 0.17 (depression) assuming a standard deviation of 0.3, as the standard deviation in an injured population may be larger than that in the general population. This is illustrated in Table 2.

**Table 2:** Sample size estimations based on the prevalence of common psychological disorders at 12 months post injury

Psychological disorder	**Prevalence at 12 months**[16]	Difference in EQ5D assume SD = 0.23, N = 456	Difference in EQ5D assume SD = 0.3, N = 456
Depression	6%	0.127	0.166

PTSD	17%	0.081	0.105

Anxiety	19%	0.077	0.101

Travel phobia	16%	0.083	0.108

#### Time scale

Participants will be recruited from June 2010 to June 2012.

## Discussion

This will be the first UK study to provide detailed estimates of the prevalence of psychological morbidities following a wide range of injuries in working age adults, and to assess their effect on functioning and health and social care resource use. It will use a range of validated standardised outcome measures and unlike many previous studies, will not rely solely on the use of screening tools for measuring psychological morbidity but will use the SCID to make psychiatric diagnoses. Measurement of physical, social and occupational functioning will allow an assessment of the contribution of psychological morbidity to delayed or sub-optimal recovery. The economic analysis will allow quantification of the health and social care costs and the contribution of psychological morbidity to those costs. Such information is vital if services are to be further developed to maximise recovery post injury. The nested qualitative study is a unique addition to previous quantitative studies of psychological morbidity post injury and the experiences of those with injuries, their carers and service providers will provide valuable insights into service development.

## Funding Source

This paper presents independent research commissioned by the National Institute for Health Research (NIHR) as part of the Collaboration for Leadership in Applied Health Research and Care – Nottinghamshire, Derbyshire and Lincolnshire (CLAHRC-NDL). The views expressed are those of the author(s) and not necessarily those of the NHS, the NIHR or the Department of Health.

## Competing interests

The authors declare that they have no competing interests.

## Authors' contributions

DK is the chief investigator for the study and principal investigator (PI) at the Nottingham site, NC is PI at the Surrey site, AM is PI at the Leicester/Loughborough site and ET is PI at the Bristol site. DK drafted the original grant proposal, drafted the study protocol and helped write this paper. CO produced the first draft of this paper. MA, CC, NC, AM, RM, ET contributed to writing the grant proposal. CC and CQ wrote the analysis plan that contributed to the protocol. SJ, SR, RM advised on outcome measurement tools for psychological and psychiatric disorders. MA, CP, ER, JS and MB drafted the protocol for the nested qualitative study. All study group members contributed to the protocol in terms of local implementation, recruitment strategies, working in the emergency department and inpatient settings or maximising follow-up. All authors contributed to writing and approving this paper.

## The Impact of Injuries Study Group

Nottingham: Isabel Allwood, Mark Avis, Marcus Barker, Julie Clarkson, Frank Coffey, Chris Craig, Tracey George, Trevor Jones, Stephen Joseph, Denise Kendrick, Blerina Kellezi, Richard Morriss, Julie Moss, Claire O'Brien, Cecily Palmer, Shireen Patel, Stephen Regel, Emma Rowley.

Surrey: Nicola Christie, Sarah Earthy, Sara Quinn, Jude Sleney.

Bristol: Kate Beckett, Georgina Elder, Elizabeth Towner.

Leicester/Loughborough: Emma Alderman, Jo Barnes, Andrew Morris.

## Pre-publication history

The pre-publication history for this paper can be accessed here:

http://www.biomedcentral.com/1471-2458/11/963/prepub
